# The Role of miRNAs in the Differential Diagnosis of Alzheimer’s Disease and Major Depression: A Bioinformatics-Based Approach

**DOI:** 10.3390/ijms26178218

**Published:** 2025-08-24

**Authors:** Gözde Öztan, Halim İşsever, Tuğçe İşsever

**Affiliations:** 1 Department of Medical Biology, Istanbul Faculty of Medicine, Istanbul University, Topkapı, 34093 Istanbul, Turkey; 2 Department of Public Health, Istanbul Faculty of Medicine, Istanbul University, Topkapı, 34093 Istanbul, Turkey; hissever@istanbul.edu.tr; 3 Turkish Health Institutes Presidency (TUSEB), 34718 Istanbul, Turkey; tugceissever@gmail.com

**Keywords:** Alzheimer’s disease, major depressive disorder, circulating microRNA biomarkers

## Abstract

Alzheimer’s disease (AD) and major depressive disorder (MDD) are prevalent central nervous system (CNS) disorders that share overlapping symptoms but differ in underlying molecular mechanisms. Distinguishing these mechanisms is essential for developing targeted diagnostic and therapeutic strategies. In this study, we integrated multi-tissue transcriptomic datasets from brain and peripheral samples to identify differentially expressed microRNAs (miRNAs) in AD and MDD. Functional enrichment analyses (KEGG, GO) revealed that dysregulated miRNAs in AD were associated with MAPK, PI3K–Akt, Ras, and PD-1/PD-L1 signaling, pathways linked to synaptic plasticity, neuroinflammation, and immune regulation. In contrast, MDD-associated miRNAs showed enrichment in Hippo signaling and ubiquitin-mediated proteolysis, implicating altered neurogenesis and protein homeostasis. Network analysis highlighted key disease- and tissue-specific miRNAs, notably hsa-miR-1202 and hsa-miR-24-3p, with potential roles in neuronal survival and molecular network regulation. These findings suggest that miRNAs may serve as non-invasive biomarkers for diagnosis, prognosis, and treatment monitoring in both disorders. While therapeutic targeting of miRNAs offers promise, challenges such as blood–brain barrier penetration and tissue-specific delivery remain. This integrative approach provides a translational framework for advancing miRNA-based strategies in CNS disease research.

## 1. Introduction

Alzheimer’s disease (AD) and major depressive disorder (MDD) are prevalent neuro-psychiatric conditions that often present overlapping clinical features, particularly in cognitive decline and mood disturbances. Growing evidence suggests that this clinical overlap may reflect shared neurobiological mechanisms and a substantial, though complex, genetic relationship between the two disorders [1]. This symptomatic convergence complicates accurate diagnosis and may lead to suboptimal treatment strategies. Therefore, identifying reliable biomarkers that can distinguish between AD and MDD is crucial for improving diagnostic precision and therapeutic outcomes. Recent bioinformatics analyses have highlighted several shared immune-related genes and pathways, underscoring the potential of integrative molecular approaches to differentiate these conditions [2].

MicroRNAs (miRNAs), small non-coding RNA molecules approximately 22 nucleotides in length, have emerged as significant regulators of gene expression at the post-transcriptional level. They achieve this regulation mainly through translational repression and mRNA destabilization, impacting a wide range of biological processes [3]. Importantly, miRNAs play crucial roles in neuronal development, synaptic plasticity, and neuroinflammatory pathways—mechanisms directly relevant to the pathophysiology of neurodegenerative and neuropsychiatric disorders such as AD and MDD. Their dysregulation can disrupt neural circuitry and cognitive processes, underscoring their potential as diagnostic biomarkers and therapeutic targets [4,5]. Altered miRNA expression profiles have been implicated in the progression of these disorders, suggesting their potential as diagnostic biomarkers [6]. Specifically, circulating levels of miR-125b and miR-181c were significantly down-regulated in AD patients, highlighting their role in disease mechanisms. These findings support the notion that serum-based miRNA signatures could provide a cost-effective, rapid, and noninvasive approach for early diagnosis and monitoring of AD [7].

In AD, specific miRNAs such as miR-132 and miR-212 are found to be dysregulated, affecting pathways related to amyloid-beta production and tau phosphorylation, which are hallmark features of the disease. Notably, downregulation of the miR-132/212 cluster occurs early in disease progression and shows a strong correlation with increased Tau pathology. These findings highlight the crucial role of this miRNA cluster in modulating neurodegenerative processes and its potential as a therapeutic target [8]. Similarly, in MDD, miRNAs like miR-1202 and miR-124 have been associated with neuroplastic changes and stress response mechanisms. Notably, miR-1202 regulates the expression of the metabotropic glutamate receptor 4 (mGluR4), influencing glutamatergic transmission and predicting antidepressant response [9]. Furthermore, miR-124 has been shown to modulate synaptic plasticity and the HPA axis, contributing to the regulation of neurogenesis and stress-induced neuronal remodeling in depression [10]. Furthermore, neuroinflammatory pathways, which are increasingly recognized as shared pathophysiological mechanisms in both disorders, have been linked to miRNA-mediated regulation of cytokines and immune signaling molecules. For instance, miRNAs can modulate NF-κB and NLRP3 inflammasome activation, thereby influencing the production of pro-inflammatory cytokines such as IL-1β and TNF-α. These regulatory effects highlight the therapeutic potential of targeting miRNA-driven neuroinflammatory cascades in neurodegenerative and neuropsychiatric diseases [11].

Recent studies suggest that depression may act as both a prodromal symptom and a risk factor for AD, further blurring the diagnostic boundaries between the two disorders. Notably, late-life depression has been shown to correlate with increased amyloid-beta deposition and tau pathology, which are hallmark features of AD [12].

In patients with late-life depression (LLD), circulating levels of hsa-miR-184 were found to be significantly downregulated. Knockdown of miR-184 in *Drosophila melanogaster* resulted in decreased locomotor activity and impaired memory performance, indicating a role in age-associated cognitive decline. These findings suggest that miR-184 dysregulation in LLD may contribute to molecular mechanisms relevant to AD pathology [13].

Furthermore, in the prefrontal cortex of individuals with LLD, several differentially expressed microRNAs—such as miR-124-3p and miR-9-5p—were identified, both of which are involved in regulating synaptic function and neuronal development. These miRNAs were enriched in pathways related to Wnt signaling, neurogenesis, and synaptic plasticity, all of which are essential processes implicated in cognitive resilience and AD. The observed miRNA alterations may therefore reflect early neuropathological changes shared between LLD and AD [14].

Moreover, other studies have identified several miRNAs as potential biomarkers linking LLD to neurodegenerative processes. These miRNAs modulate key molecular pathways, including neuroinflammation, neurotrophic signaling, and neuronal plasticity. Dysregulation of these pathways may increase vulnerability to cognitive decline and facilitate progression from LLD to mild cognitive impairment and ultimately to AD [15].

Taken together, the convergence of dysregulated miRNAs in LLD on key biological processes—such as synaptic remodeling, hippocampal neurogenesis, and immune activation—supports a mechanistic overlap with early AD pathology [13,14,15]. Rather than reflecting an isolated affective disorder, LLD may constitute a prodromal phase within the broader neurodegenerative continuum [15]. miRNAs such as miR-124 and miR-184, due to their roles in regulating memory, synaptic integrity, and neuronal adaptation, may serve not only as biomarkers but also as active molecular mediators driving the progression from LLD to mild cognitive impairment and eventually to AD [13,14]. This perspective underscores the importance of miRNA-based profiling in LLD as a means of identifying individuals at elevated risk for subsequent neurodegenerative decline [15].

Additionally, individuals with a history of depression have a significantly higher likelihood of developing AD, underscoring the need for early screening and integrated management approaches [16]. This bidirectional relationship underscores the necessity of identifying molecular tools that can distinguish the early pathological processes unique to each condition. In this context, miRNAs emerge not only as passive biomarkers but also as active players in the neurodegenerative cascade, modulating key elements of neuronal integrity, neurogenesis, and synaptic remodeling. Recent findings highlight that dysregulated miRNAs can both reflect and drive disease progression through pathways affecting synaptic function and neuroinflammation [17]. Moreover, novel structural insights into ion channel modulation suggest that targeting these processes may offer precise intervention strategies for halting neurodegenerative decline [18].

Furthermore, miRNAs are present in a variety of biofluids, including serum, plasma, cerebrospinal fluid (CSF), and saliva, making them ideal candidates for non-invasive biomarker discovery. Their stability in circulation and specificity to cell type or disease state enhance their translational value for clinical application. Notably, endogenous plasma miRNAs remain stable even after multiple freeze–thaw cycles or prolonged room temperature incubation, demonstrating exceptional resilience [19]. Additionally, cell-free miRNAs can be packaged within exosomes or bound to proteins such as Argonaute 2, which further protects them from degradation and supports their reliable detection in clinical samples [20]. In addition to diagnosis, miRNAs are increasingly explored as potential therapeutic targets through miRNA mimics or inhibitors designed to restore aberrant expression patterns. Notably, recent strategies include the development of locked nucleic acid (LNA) anti-miRs and synthetic mimics that enhance stability and targeting specificity in vivo [21]. Moreover, combination approaches employing miRNA-based therapeutics with conventional drugs have shown synergistic effects, highlighting their promise for integrated cancer therapy [22].

Despite these advances, most current studies investigating miRNA roles in AD and MDD have focused on isolated conditions. Comparative analyses that explore differential expression patterns across both disorders in parallel are limited. Therefore, integrative bioinformatic approaches using publicly available datasets offer a promising strategy to uncover unique and shared molecular signatures with diagnostic relevance.

This study builds on this rationale by applying differential expression analysis and downstream bioinformatic enrichment to identify miRNAs with potential discriminative value between AD and MDD. The findings aim to provide a molecular framework for refining diagnostic accuracy, informing clinical decision-making, and ultimately guiding personalized medicine initiatives in neuropsychiatry.

## 2. Results

### 2.1. miRNA Profiling in the Temporal Cortex of AD Patients

Following differential expression analysis of the GSE157239 dataset [23]—which includes post-mortem temporal cortex tissue samples from 8 individuals diagnosed with AD and 8 age-matched cognitively healthy controls—a panel of the top 10 upregulated and top 10 downregulated miRNAs was identified based on log2 fold change values and an adjusted *p*-value threshold of <1 (Table 1). The analysis was conducted using the Affymetrix^®^ miRNA 4.1 microarray platform, which targets mature and precursor human miRNAs annotated in miRBase v20 [24]. The most upregulated miRNAs—such as hsa-miR-1299, hsa-miR-1202, and hsa-miR-4492—may reflect neuroinflammatory processes and glial activation, whereas downregulated miRNAs like hsa-miR-4286, hsa-miR-3651, and hsa-miR-664b-3p are potentially involved in neuronal maintenance, synaptic regulation, and neuroimmune signaling. These differentially expressed brain-derived miRNAs offer valuable insights into the molecular alterations occurring within the central nervous system during AD pathogenesis and may guide further functional validation and biomarker discovery efforts [25].

**Figure 1:**
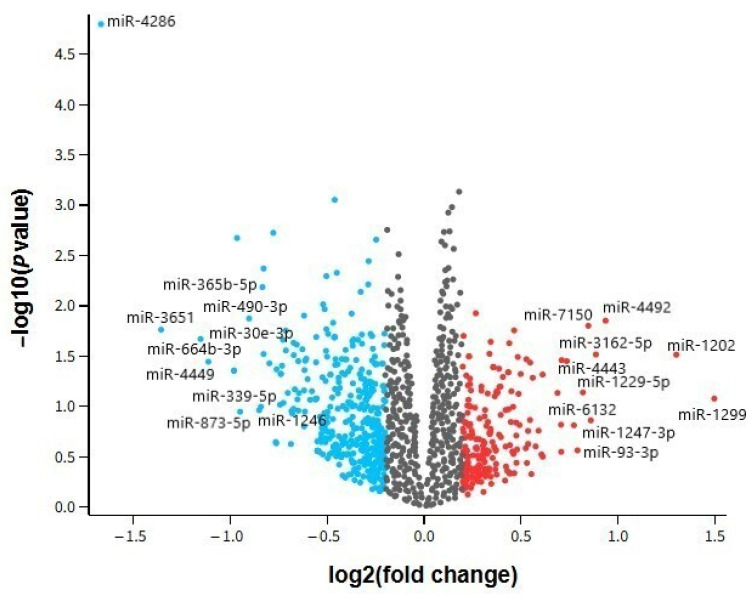
Volcano plot illustrating differentially expressed miRNAs in the temporal cortex of AD patients versus cognitively normal controls (GSE157239). Each dot represents one miRNA. Red points indicate significantly up-regulated miRNAs and blue points indicate significantly down-regulated miRNAs; gray points denote miRNAs that did not meet the significance thresholds (|log2FC| ≥ 0.5 and *p* < 0.05).

To visualize the distribution and significance of differentially expressed miRNAs identified in the GSE157239 dataset, a volcano plot was generated. This graphical representation enables the simultaneous assessment of both the magnitude of expression change (log_2_ fold change) and statistical significance (−log_10_ *p*-value) for each miRNA. Based on the cut-off thresholds of |log_2_ fold change| ≥ 0.2 and significance level < 1, several miRNAs were found to be notably dysregulated in the temporal cortex of individuals with AD compared to cognitively normal controls. Upregulated miRNAs such as hsa-miR-1299, hsa-miR-1202, and hsa-miR-4492 are highlighted on the right side of the plot, while hsa-miR-4286, hsa-miR-3651, and hsa-miR-664b-3p are among the prominent downregulated candidates on the left. This visualization underscores the asymmetry in miRNA expression patterns and highlights key candidates for further mechanistic and biomarker research in AD (Figure 1).

### 2.2. Circulating miRNA Signatures in AD (GSE120584 Dataset)

Following differential expression analysis of the GSE120584 dataset [26]—which includes serum samples from 1015 AD patients and 288 cognitively normal controls—the top 10 upregulated and top 10 downregulated miRNAs were identified based on log2 fold change values and an adjusted *p*-value threshold of <1 (Table 2). The most upregulated miRNAs include hsa-miR-208a-5p, hsa-miR-6761-3p, and hsa-miR-3646, which may be implicated in neuroinflammation and systemic responses to AD pathology. Conversely, downregulated miRNAs such as hsa-miR-125a-3p, hsa-miR-6131, and hsa-miR-24-3p may reflect disruptions in synaptic plasticity, neuronal survival, and neuroimmune balance. These interpretations align with the recognized role of circulating miRNA signatures as non-invasive biomarkers in AD [27,28] and broader literature on miRNA-mediated regulation in neurodegeneration [29].

**Figure 2:**
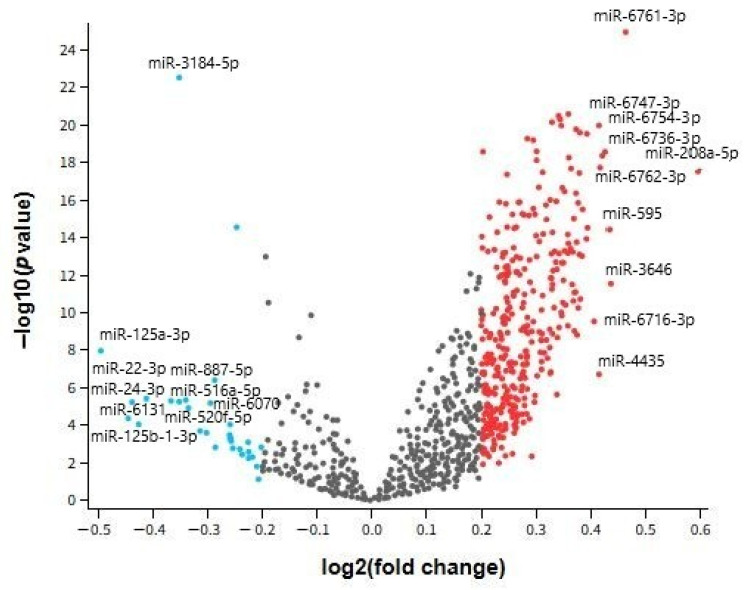
Volcano Plot representation of miRNA expressions obtained from the Alzheimer’s disease group in the GSE120584 dataset.

The volcano plot depicted in Figure 2 provides a comprehensive visualization of differentially expressed miRNAs in serum samples from individuals with AD compared to healthy controls, based on the GSE120584 dataset. Each point on the plot represents a single miRNA, with the x-axis showing the log2 fold change and the y-axis indicating the −log10 adjusted *p*-value. This graphical representation enables rapid identification of miRNAs exhibiting both statistically significant and biologically meaningful expression differences. Highlighted miRNAs with positive log2 fold changes—such as hsa-miR-208a-5p and hsa-miR-6761-3p—are strongly upregulated in AD, whereas those with negative values—such as hsa-miR-125a-3p and hsa-miR-24-3p—are markedly downregulated. The volcano plot not only supports the quantitative results from Table 2 but also underscores the distinct expression profile of circulating miRNAs in AD, paving the way for biomarker discovery and further functional exploration.

### 2.3. Peripheral Blood–Derived miRNA Signatures in Major Depression (GSE81152 Dataset)

The GSE81152 dataset [30] comprises whole-blood microRNA profiles from 61 patients diagnosed with MDD and 19 healthy controls. In the original study, participants were later treated with either electroconvulsive therapy or ketamine to assess treatment-related expression changes. However, the dataset also includes baseline miRNA profiles prior to treatment, which were analyzed to identify differentially expressed miRNAs between the MDD and control groups. Based on GEO2R analysis, the top 10 upregulated and top 10 downregulated miRNAs—ranked by fold change—are listed in Table 3.

Following differential expression analysis, a panel of the top 10 upregulated and top 10 downregulated miRNAs in MDD samples was identified based on log2 fold change values (Table 3). Among the upregulated miRNAs, hsa-miR-4445-3p, hsa-miR-5579-5p, and hsa-miR-3129-3p were particularly prominent and have been associated with cellular stress responses and immune signaling. Conversely, downregulated miRNAs such as hsa-let-7b-5p, hsa-miR-539-3p, and hsa-miR-1185-2-3p may reflect disrupted neuronal plasticity and energy metabolism. Moreover, evidence supports the idea that circulating miRNAs have utility as peripheral molecular indicators of MDD pathophysiology [31].

**Figure 3:**
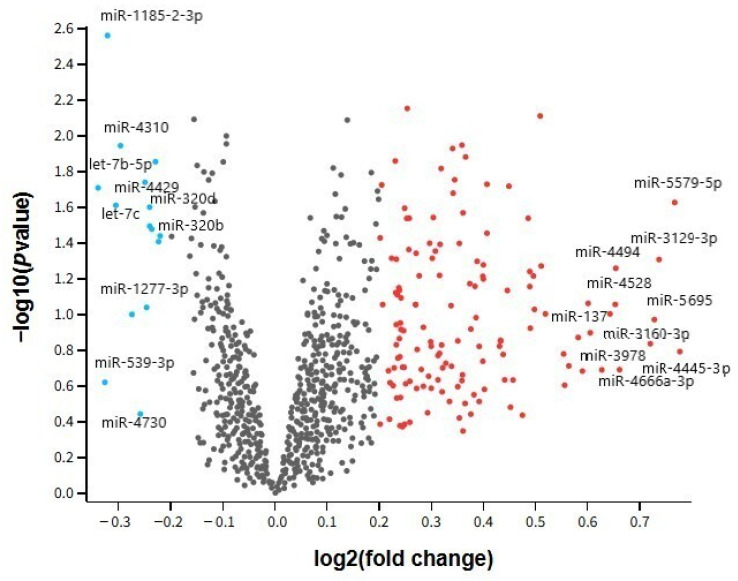
Volcano plot showing differential expression of miRNAs between MDD patients and healthy controls in the GSE81152 dataset (whole blood samples).

The volcano plot presented in Figure 3 illustrates the overall distribution of differentially expressed miRNAs in whole blood samples from individuals with MDD compared to healthy controls, based on the GSE81152 dataset. Each point corresponds to a single miRNA, with the x-axis representing the log_2_ fold change and the y-axis denoting the −log_10_ adjusted *p*-value. This visualization effectively highlights miRNAs exhibiting the most pronounced expression shifts—both upregulation and downregulation—between the groups. Upregulated miRNAs such as hsa-miR-4445-3p and hsa-miR-5579-5p, along with downregulated candidates like hsa-miR-1185-2-3p and hsa-let-7b-5p, are clearly distinguishable, reinforcing their potential role as peripheral molecular markers in MDD pathophysiology. Furthermore, the volcano plot underscores the global expression landscape and variability among miRNAs, offering visual support for the differential expression analysis and aiding in the prioritization of candidates for future validation studies [32].

### 2.4. Brain Tissue–Derived miRNA Signatures in Major Depression (GSE58105 Dataset)

Following differential expression analysis of the GSE58105 dataset [9]—comprising ventrolateral prefrontal cortex samples from 14 individuals with MDD and 11 healthy controls—the top 10 upregulated and top 10 downregulated miRNAs were identified based on log2 fold change values (Table 4).

Among the upregulated miRNAs, hsa-miR-1281, hsa-miR-1825, and hsa-miR-451 stood out, potentially indicating immune-related or metabolic alterations in the central nervous system. Conversely, downregulated candidates such as hsa-miR-1202, hsa-miR-575, and hsa-miR-1225-5p are implicated in pathways associated with synaptic signaling and mood regulation. Notably, miR-1202—a brain-enriched and primate-specific miRNA—has been experimentally validated in the original study and shown to regulate the mGluR4, a key molecule in antidepressant response. These results emphasize the biological relevance of centrally dysregulated miRNAs in the molecular pathophysiology of MDD [9]. 

**Figure 4:**
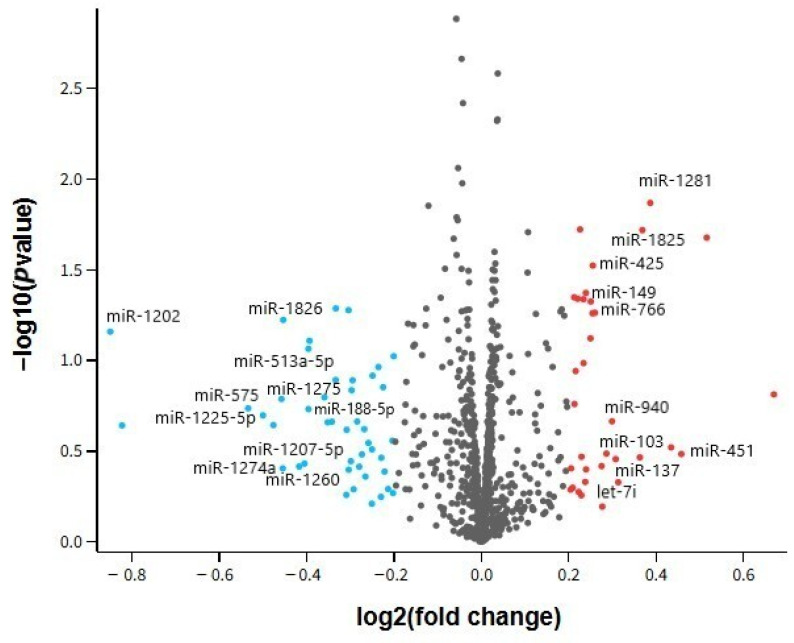
Volcano Plot representation of miRNA expression profiles obtained from the ventrolateral prefrontal cortex in the major depression group, based on the GSE58105 dataset.

The volcano plot presented in Figure 4 visualizes the distribution of miRNA expression changes in prefrontal cortex samples of MDD patients compared to healthy controls, based on the GSE58105 dataset. Each dot represents a distinct miRNA, plotted according to its log2 fold change (x-axis) and −log10 adjusted *p*-value (y-axis). The analysis was performed using a significance level cut-off of 1 and a log2 fold change threshold of ±0.2 to allow a broader assessment of expression patterns. While no miRNAs met stringent statistical thresholds (e.g., adjusted *p* < 0.05), the plot highlights general trends in up- and downregulation. Upregulated miRNAs such as hsa-miR-1281 and hsa-miR-1825 are located on the right, whereas downregulated miRNAs including hsa-miR-1202 and hsa-miR-575 appear on the left. These shifts may still reflect relevant biological alterations, especially when supported by previous findings.

### 2.5. Comparative Analysis of Differentially Expressed miRNAs Across AD and MDD Datasets

To provide a comparative overview of miRNA alterations across AD and MDD, differentially expressed miRNAs identified in each dataset were systematically examined. Table 5 summarizes selected miRNAs that were found to be differentially expressed in at least one of the datasets included in this study. Notably, hsa-miR-24-3p demonstrated consistent downregulation across all tissues analyzed—including serum, blood, and brain—suggesting a robust association with neuropsychiatric pathology [33]. Other miRNAs, such as hsa-miR-125a-3p, hsa-miR-664b-3p, and hsa-miR-4443, displayed expression changes in more than one tissue but were not consistently regulated across all datasets. Importantly, expression directionality was not always conserved across tissues; for instance, some miRNAs were upregulated in brain samples but downregulated in peripheral fluids, or vice versa [34]. These tissue-dependent discrepancies underscore the necessity of cautious interpretation when identifying shared miRNA signatures and reinforce the importance of validating candidate biomarkers within specific tissue contexts before assuming systemic concordance [35,36].

### 2.6. Functional Enrichment (GO/KEGG) Findings

This study performed Gene Ontology (GO) and KEGG pathway enrichment analyses to identify the biological processes and molecular pathways potentially regulated by the top 20 differentially expressed miRNAs in AD and MDD. The top 10 GO and KEGG terms for each condition are summarized in Table 6.

In the AD group, GO analysis revealed that most enriched terms were related to nuclear localization and nucleic acid interactions. Specifically, protein binding, RNA binding, nucleus, and nucleoplasm emerged as the most significantly enriched terms. These findings suggest widespread miRNA involvement in post-transcriptional regulation and chromatin-associated functions in AD. KEGG analysis further indicated enrichment in cancer- and inflammation-associated pathways, such as Cell cycle, PI3K-Akt signaling pathway, Pathways in cancer, and Focal adhesion, aligning with known cellular stress responses and neurodegenerative mechanisms in AD.

Similarly, MDD-associated miRNAs were enriched in GO terms such as protein binding, RNA binding, chromatin binding, and viral process, indicating functional overlaps with transcriptional regulation and immune-related signaling. KEGG pathway enrichment in MDD highlighted Hippo signaling, Proteoglycans in cancer, Ubiquitin-mediated proteolysis, and Alzheimer disease pathways, suggesting convergence between psychiatric and neurodegenerative disorders at the molecular level (Table 6).

These expanded analyses allow more nuanced comparisons and support the biological plausibility of both shared and disease-specific miRNA-mediated regulatory mechanisms across AD and MDD.

**Figure 5:**
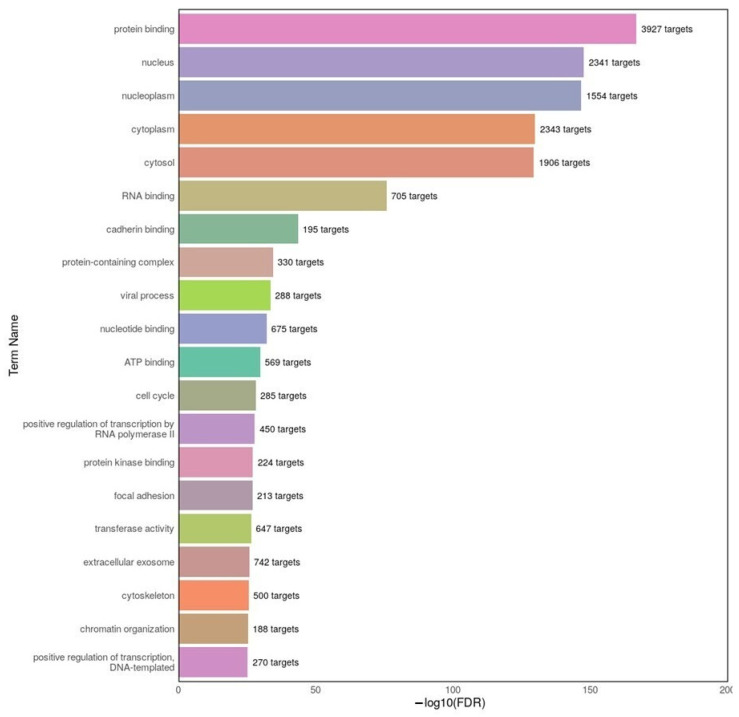
Top 20 GO terms enriched among the predicted target genes of differentially expressed miRNAs in the AD group. GO enrichment analysis was performed using DIANA-miRPath v4.0 based on the top 20 differentially expressed miRNAs (10 upregulated and 10 downregulated). The x-axis represents the −log10(FDR) values, indicating the statistical significance of each term. The number of target genes associated with each GO term is shown at the end of the corresponding bar. The results highlight key biological processes, cellular components, and molecular functions potentially affected by miRNA dysregulation in AD.

To better understand the biological implications of the dysregulated miRNAs in AD, a GO enrichment analysis was conducted using DIANA-miRPath v4.0, focusing on the top 20 differentially expressed miRNAs identified in the AD datasets. The results (Figure 5) revealed that the predicted targets of these miRNAs were significantly associated with a variety of key biological categories. Among the top enriched terms were protein binding, nucleus, cytoplasm, RNA binding, and nucleotide binding, suggesting that miRNA dysregulation in AD may influence essential cellular processes, particularly those involved in gene expression regulation, intracellular transport, and macromolecular interactions. The enrichment of terms such as cell cycle, positive regulation of transcription by RNA polymerase II, and focal adhesion further supports the hypothesis that altered miRNA activity may contribute to neuronal dysfunction through broad regulatory mechanisms. These findings underscore the potential of miRNA-targeted pathways as biomarkers or therapeutic avenues in AD.

**Figure 6:**
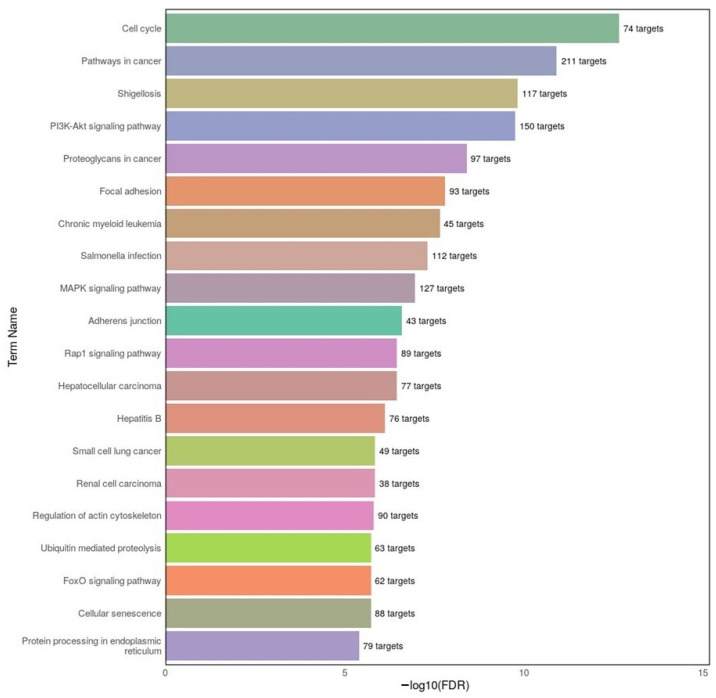
Top 20 KEGG pathways enriched among the predicted target genes of differentially expressed miRNAs in the AD group. The analysis was performed using DIANA-miRPath v4.0 with the top 20 dysregulated miRNAs. The x-axis represents the −log10(FDR) value indicating statistical significance, while the number of associated target genes is displayed at the end of each bar. Pathways such as cell cycle, PI3K-Akt signaling, and MAPK signaling highlight potentially altered signaling cascades in AD pathogenesis.

To further elucidate the functional impact of miRNA dysregulation in AD, KEGG pathway enrichment analysis was conducted using DIANA-miRPath v4.0 based on the top 20 miRNAs (10 upregulated and 10 downregulated). The results are visualized in Figure 6 and demonstrate significant involvement of miRNA targets in several critical signaling pathways. Prominent among these were cell cycle, PI3K-Akt signaling pathway, MAPK signaling, and focal adhesion, all of which are implicated in neuronal survival, plasticity, and inflammation—hallmarks of AD pathology. Additionally, several cancer-related pathways (pathways in cancer, proteoglycans in cancer, chronic myeloid leukemia) were enriched, consistent with the overlap in molecular mechanisms between neurodegenerative and proliferative diseases. These findings reinforce the multifaceted role of miRNAs in regulating diverse biological processes that may contribute to the complex molecular landscape of AD.

**Figure 7:**
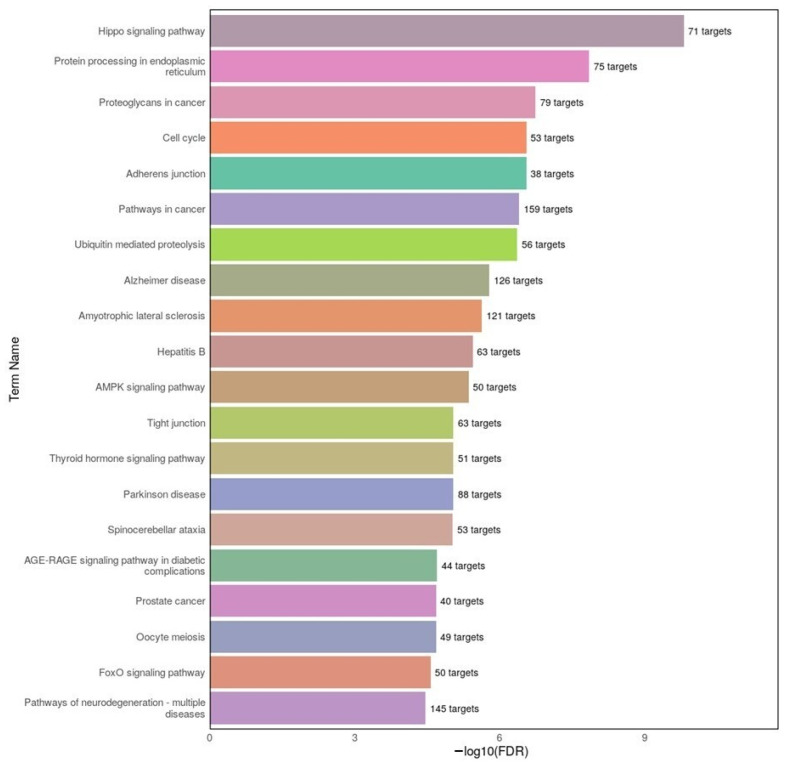
Top 20 KEGG pathways enriched among predicted target genes of differentially expressed miRNAs in the MDD group. The analysis was conducted using DIANA-miRPath v4.0 based on the top 20 miRNAs. The x-axis represents the −log10(FDR) significance score, while the number of target genes associated with each pathway is shown on the right side of the bars. Enriched pathways include Alzheimer disease, Parkinson disease, AMPK signaling, and ubiquitin-mediated proteolysis, suggesting shared neurodegenerative and cellular stress-related mechanisms in MDD.

To explore the biological functions of differentially expressed miRNAs in MDD, KEGG pathway enrichment analysis was performed using DIANA-miRPath v4.0. The top 20 pathways based on −log10(FDR) scores are illustrated in Figure 7. Several neurodegenerative disease-associated pathways, including Alzheimer disease, Parkinson disease, and amyotrophic lateral sclerosis, were significantly enriched, indicating potential overlap in molecular mechanisms between MDD and neurodegeneration. Moreover, signaling pathways such as Hippo, AMPK, and FoxO, which are involved in cellular homeostasis, stress responses, and neuroplasticity, were prominently represented. Pathways related to ubiquitin-mediated proteolysis and protein processing in the endoplasmic reticulum further suggest involvement of impaired protein clearance mechanisms in MDD. These results support the hypothesis that miRNA dysregulation in MDD may modulate key signaling cascades relevant to both neuropsychiatric and neurodegenerative processes.

**Figure 8:**
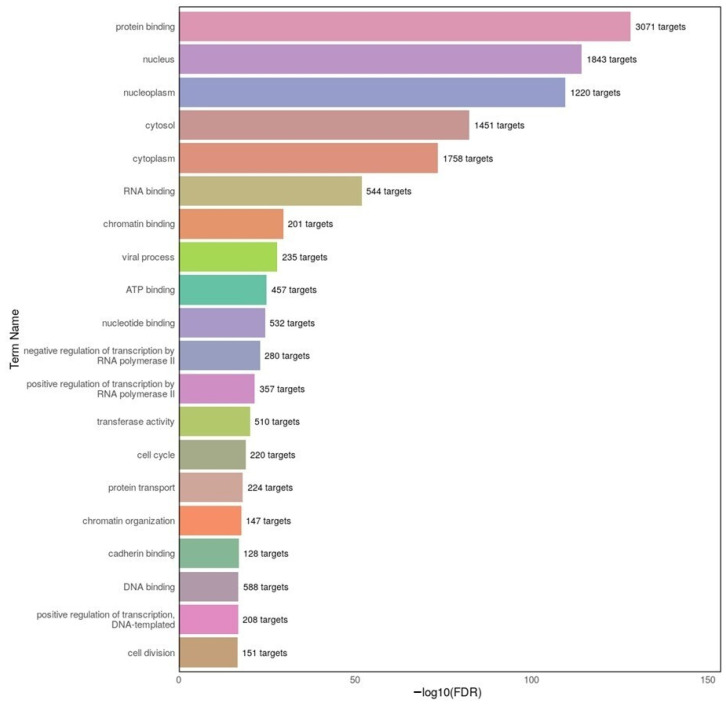
Top 20 GO terms enriched among the predicted target genes of the top 20 differentially expressed miRNAs in the MDD group. The GO analysis was performed using DIANA-miRPath v4.0. The x-axis represents the −log10(FDR) significance score, and the number of predicted gene targets associated with each term is shown within the bars. The top terms include protein binding, nucleus, cytoplasm, and RNA binding, reflecting core regulatory processes potentially influenced by miRNA dysregulation in MDD.

To gain insight into the biological processes and molecular functions potentially affected by differentially expressed miRNAs in MDD, GO enrichment analysis was conducted via DIANA-miRPath v4.0. The results are presented in Figure 8 and reflect the top 20 enriched GO terms based on the predicted targets of the top 20 miRNAs. Dominant terms include protein binding, nuclear localization, RNA binding, cytoplasmic processes, and ATP binding, nucleotide binding, indicating a strong association with transcriptional regulation, RNA metabolism, and intracellular transport. Additionally, terms such as chromatin organization, cell cycle, and viral process suggest involvement in epigenetic modulation and cellular stress responses. Collectively, these findings highlight the potential of miRNA-mediated regulation in shaping diverse molecular mechanisms underlying MDD pathophysiology.

### 2.7. Integrated miRNA–mRNA–Pathway Network

**Figure 9:**
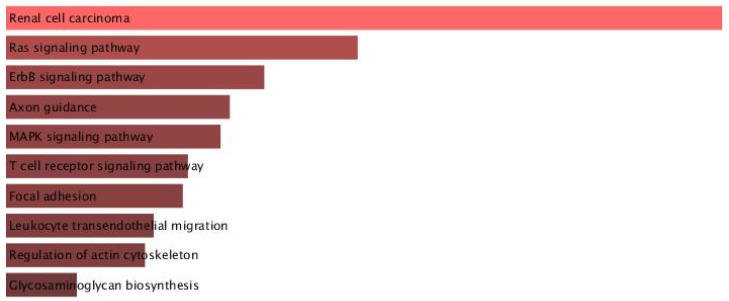
KEGG enrichment analysis of hsa-miR-1202 target genes (miRDB score ≥ 80) using the Enrichr platform, showing the top 10 significantly enriched pathways.

Among the differentially expressed miRNAs in AD and MDD, hsa-miR-1202 was identified as a shared miRNA. To evaluate its potential regulatory role, target gene prediction was performed using miRDB (http://mirdb.org; accessed on 6 August 2025), yielding 81 target genes with a prediction score of ≥80. These predicted targets were subsequently analyzed for functional enrichment using the KEGG 2021 Human database via the Enrichr platform (https://maayanlab.cloud/Enrichr; accessed on 6 August 2025). The enrichment analysis revealed significant associations with multiple biological pathways, including renal cell carcinoma, Ras signaling, ErbB signaling, axon guidance, MAPK signaling, T cell receptor signaling, focal adhesion, leukocyte transendothelial migration, regulation of actin cytoskeleton, and glycosaminoglycan biosynthesis (Figure 9) (Supplementary Table S1).

**Figure 10:**
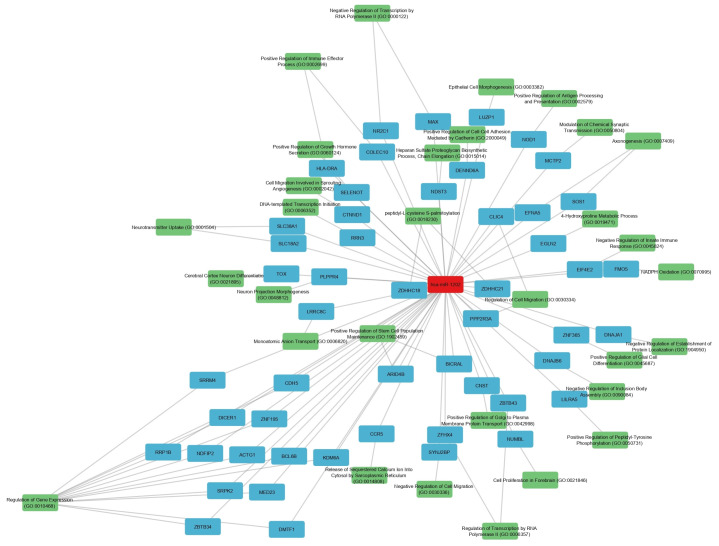
Integrated hsa-miR-1202–gene–GO Biological Process network.

To further dissect the functional context, these 81 genes were assessed for enrichment in GO Biological Process terms. After filtering for significance (*p* < 0.05), 51 genes remained. For each gene, the GO term with the lowest *p*-value was selected to represent its primary functional annotation. These pathways collectively implicate hsa-miR-1202 in processes related to intracellular signaling, immune regulation, and neural connectivity, as further illustrated by the constructed network (Figure 10).

The network displays 83 nodes (1 miRNA, 51 genes, and 31 GO IDs) and 102 edges. Nodes are colored by type: hsa-miR-1202 (red), genes (blue), and GO terms (green). Node sizes are proportional to −log10(p), highlighting genes and GO terms with stronger statistical support. Inhibitory miRNA→gene edges (red, blunt-ended) indicate predicted repression of target genes, while GO→gene edges (gray, arrow-headed) connect each gene to its most significantly enriched GO Biological Process term (*p* < 0.05). The layout separates the miRNA, gene, and GO clusters, revealing functional groupings such as transcriptional regulation, neuronal differentiation, immune modulation, and membrane trafficking. GO term labels are shown as GO IDs for clarity; full descriptions are provided in Table 7.

The final hsa-miR-1202 network comprised 83 nodes (1 miRNA, 51 genes, and 31 GO IDs displayed) and 102 edges. Node sizes in the network are proportional to −log10(p), emphasizing genes and GO terms with stronger statistical support. The layout distinctly separates the miRNA, gene, and GO clusters, revealing coherent functional groups, including transcriptional regulation, neuronal differentiation, immune modulation, and membrane trafficking. Inhibitory miRNA→gene edges are shown in red with blunt ends, reflecting the canonical repressive role of miRNAs, while GO→gene edges are shown in gray with arrowheads, indicating functional annotation. Table 7 lists the 51 predicted target genes of hsa-miR-1202, their most significantly associated GO terms, and corresponding statistical values. GO terms in Figure 10 are abbreviated to GO IDs for clarity; full term descriptions are retained in Table 7 along with *p*-values and their −log10 transformations, which were used for proportional node sizing in the visualization.

## 3. Discussion

The present study investigated microRNA (miRNA) expression profiles in AD and MDD across both central (cortical tissue) and peripheral (serum, whole-blood) compartments using publicly available Gene Expression Omnibus datasets. We identified a panel of 10 overlapping miRNAs between the disorders, with particular emphasis on hsa-miR-24-3p and hsa-miR-1202, and performed functional enrichment and network analyses to explore their potential mechanistic roles. Below, our findings are interpreted in light of existing literature, highlighting concordances and discrepancies, possible biological underpinnings, and clinical implications.

### 3.1. Overall Interpretation of Differentially Expressed miRNAs in AD and MDD

In our analysis, several miRNAs demonstrated dysregulation in both AD and MDD, suggesting shared molecular pathways despite their distinct clinical phenotypes. Notably, hsa-miR-24-3p exhibited consistent downregulation across all examined tissues, while hsa-miR-1202 displayed contrasting patterns—upregulated in AD brain tissue but downregulated in MDD cortex. Other miRNAs, such as hsa-miR-125a-3p and hsa-miR-664b-3p, were altered in multiple datasets but without uniform directionality.

These results are in agreement with studies reporting overlapping miRNA perturbations in neurodegenerative and psychiatric disorders [37,38]. For example, Lopez et al. [9] identified altered cortical expression of miR-1202 in MDD, while Lau et al. [8] demonstrated miRNA involvement in amyloid precursor protein processing in AD. However, unlike some previous reports that described uniform regulation of candidate miRNAs across biofluids and brain tissue, our findings revealed tissue-dependent variation, possibly reflecting compartment-specific transcriptional control or differential release mechanisms into circulation [36].

Clinically, the convergence of certain miRNAs across disorders reinforces their potential as multi-disease biomarkers, but our results also caution against assuming systemic reflection of brain changes without tissue-specific validation [39].

### 3.2. Tissue-Specific and Systemic miRNA Signatures

Our dataset comparison showed that hsa-miR-24-3p was robustly downregulated in both brain and peripheral compartments, suggesting a systemic disturbance potentially accessible for non-invasive biomarker development. In contrast, hsa-miR-1202 presented opposing regulation between AD and MDD brain samples, and several other miRNAs displayed inconsistent changes between peripheral and central samples.

This divergence aligns with reports that circulating miRNA profiles often only partially mirror CNS expression patterns [40,41]. Such differences can result from the selective packaging of miRNAs into exosomes, the influence of peripheral inflammation, or varying stability in biofluids [42]. Our finding that miR-24-3p maintained consistent downregulation across compartments strengthens its candidacy for diagnostic purposes, while the discordant patterns observed for miR-1202 emphasize the necessity of context-specific interpretation.

From a biological perspective, these tissue-dependent differences may indicate that certain miRNAs serve specialized roles within the CNS that are not replicated peripherally, or that peripheral expression is modulated by systemic factors independent of brain pathology [43,44,45].

### 3.3. Biological Significance of Shared miRNAs: Focus on hsa-miR-24-3p and hsa-miR-1202

In our study, hsa-miR-24-3p emerged as the most consistent cross-disease marker, being downregulated in all datasets. Literature supports its role in modulating neuronal apoptosis, microglial activation, and endothelial function [25,26]. Our consistent finding across brain and blood is in line with Liu et al., who reported reduced serum miR-24-3p levels in AD patients [46], and with Marchetti et al., who observed that inhibition of miR-24-3p in ischemic limb models led to dysfunctional vessel formation, supporting its role in vascular integrity [47].

hsa-miR-1202, on the other hand, showed disease- and tissue-specific regulation. Its upregulation in AD cortex may represent a compensatory mechanism to sustain glutamatergic signaling amid synaptic degeneration, while its downregulation in MDD cortex could exacerbate glutamatergic hypoactivity, a known feature of depression [9,31]. Our network analysis linking miR-1202 to MAPK, Ras, and ErbB signaling pathways supports its involvement in both synaptic plasticity and inflammatory regulation [48,49,50].

These results, when viewed alongside experimental data on antidepressant-induced normalization of miR-1202 [5], highlight its potential as both a disease-specific and treatment-responsive biomarker.

### 3.4. Functional Implications from GO and KEGG Enrichment Analyses

Our GO enrichment analysis revealed that dysregulated miRNAs in both AD and MDD target genes involved in protein binding, RNA binding, and nuclear localization. These categories suggest a strong influence on transcriptional regulation and post-transcriptional processing—echoing previous findings in the CNS context, such as the role of miR-124 in modulating alternative splicing and nuclear gene regulation in neuronal cells [43], and the broader post-transcriptional regulatory functions of miRNAs in CNS trauma and degeneration [51].

KEGG pathway analysis in our study showed significant overlap between disorders in cell cycle regulation, PI3K–Akt signaling, and focal adhesion. In AD datasets, we found enrichment of MAPK and PI3K–Akt signaling, consistent with prior studies linking these pathways to tau phosphorylation and neuroinflammation [50,52]. In MDD datasets, enrichment in Hippo signaling and ubiquitin-mediated proteolysis points toward altered neurogenesis and protein homeostasis, consistent with evidence that chronic stress activates the Hippo–YAP pathway in depression models [53], and that dysregulation of ubiquitin–proteasome subunit expression is observed in the post-mortem brains of MDD patients [54].

By directly linking our differentially expressed miRNAs to these pathways, our results not only validate earlier work but also extend it by identifying specific miRNA–pathway associations that may underlie shared and divergent aspects of AD and MDD.

### 3.5. Overlap Between Neurodegenerative and Psychiatric Pathways

Our KEGG enrichment results demonstrated that MDD-associated miRNAs also map to pathways typically associated with neurodegenerative diseases, including Alzheimer disease, Parkinson disease, and amyotrophic lateral sclerosis. This finding aligns with epidemiological evidence that depression is both a risk factor for and a prodrome of dementia [48,55].

While previous studies have suggested shared mechanisms such as mitochondrial dysfunction and oxidative stress [56,57], our data provide a direct molecular link via miRNA regulatory networks. For example, the appearance of AD pathway enrichment in MDD miRNA targets reinforces the possibility of overlapping early pathogenic events [58,59].

Clinically, these overlaps underscore the potential for shared therapeutic targets and for miRNA-based tools to aid in early detection of patients at risk for transitioning from psychiatric to neurodegenerative conditions [6,15,60].

### 3.6. Potential Clinical Applications: Biomarker and Therapeutic Prospects

Our identification of hsa-miR-24-3p as a consistently downregulated miRNA across tissues supports its candidacy as a non-invasive biomarker. Meanwhile, hsa-miR-1202’s regulation pattern and responsiveness to antidepressants make it attractive for monitoring treatment effects in MDD and potentially tracking disease progression in AD [9,61].

The use of circulating miRNAs as biomarkers is supported by their stability in biofluids and their detectability with standard molecular methods [62]. Therapeutically, targeting these miRNAs with mimics or inhibitors could theoretically restore dysregulated pathways, though delivery challenges remain—particularly for CNS diseases where the blood–brain barrier presents a major obstacle [63,64,65,66].

### 3.7. Strengths and Limitations of the Present Study

A strength of our work is the integration of multi-tissue data from both AD and MDD, enabling direct comparison of central and peripheral miRNA signatures. Our combined approach of differential expression, functional enrichment, and network analysis provided mechanistic insights beyond mere list comparisons. However, our study is limited by several factors. Heterogeneity in microarray platforms and potential batch effects could influence comparability across datasets [67].

Cross-dataset and cross-tissue comparisons in our study should be interpreted with caution. Public GEO cohorts differ in platform technology (e.g., probe content and miRBase annotation versions), sample sizes, demographic and clinical composition, tissue source (post-mortem cortex vs. whole blood/serum), RNA isolation and pre-analytical handling, and preprocessing pipelines. These factors—together with potential batch effects—can yield apparent discrepancies in the direction of change for the same miRNA across compartments [35,68]. Accordingly, our cross-condition summary prioritizes recurrence of candidates rather than assuming conserved regulation across tissues. To strengthen generalizability, future work should validate these in silico findings in well-matched, deeply phenotyped cohorts using harmonized protocols, include tissue-matched brain and biofluid specimens (e.g., EV-enriched plasma/CSF), and apply cross-platform normalization strategies [44,69]. Such designs will be essential to determine whether candidate miRNAs function as systemic biomarkers or reflect compartment-specific biology.

The cross-sectional nature of the data precludes conclusions about causality. Furthermore, our analysis was limited by the lack of access to detailed demographic and clinical information, such as sex, age, number of depressive episodes, and comorbidities, which precluded the assessment of potential moderating effects on miRNA expression patterns. Such variables are known to influence both the prevalence and molecular presentation of AD and MDD, and their absence may obscure important subgroup-specific associations [70,71]. In addition, the absence of peripheral biomarker data—particularly amyloid-beta concentrations in blood, serum, or brain tissue—restricted our ability to directly investigate mechanistic links between circulating or brain-expressed miRNAs and established pathological hallmarks. Inclusion of such data in future studies would enable more precise modeling of disease pathways and strengthen causal inference.

### 3.8. Future Research Directions

Future research should aim to validate the candidate miRNAs identified in this study—particularly hsa-miR-24-3p and hsa-miR-1202—in longitudinal cohorts to assess their predictive value for disease onset and progression. Integrating additional omics layers such as transcriptomics, proteomics, and metabolomics will provide a more comprehensive understanding of the molecular networks underlying both AD and MDD. Advanced approaches, including cell type–specific profiling and spatial transcriptomics, could help to map miRNA–mRNA interactions within distinct neural circuits, offering insights into cell-specific regulatory mechanisms. Furthermore, preclinical studies modulating the expression of these miRNAs will be crucial to evaluate their therapeutic potential and safety. Finally, combining miRNA biomarkers with neuroimaging, cognitive testing, and other clinical measures could lead to more accurate and disease-specific diagnostic tools, paving the way for precision medicine strategies in neuropsychiatric disorders.

## 4. Materials and Methods

### 4.1. Dataset Selection

Publicly available datasets were retrieved from the NCBI Gene Expression Omnibus (GEO) database (National Center for Biotechnology Information, Bethesda, MD, USA) to investigate miRNA expression profiles in AD and MDD. The initial dataset selection was revised during the study to ensure compatibility with updated research objectives and uniform analytical processing. For AD, the analysis included GSE157239 (https://www.ncbi.nlm.nih.gov/geo/query/acc.cgi?acc=GSE157239, accessed on 19 May 2025), which contains miRNA expression profiles from post-mortem prefrontal cortex tissue, and GSE120584 (https://www.ncbi.nlm.nih.gov/geo/query/acc.cgi?acc=GSE120584, accessed on 19 May 2025), which provides serum-derived miRNA data. For MDD, two datasets were selected: GSE58105 (https://www.ncbi.nlm.nih.gov/geo/query/acc.cgi?acc=GSE58105, accessed on 19 May 2025), representing miRNA profiles from ventrolateral prefrontal cortex tissue, and GSE81152 (https://www.ncbi.nlm.nih.gov/geo/query/acc.cgi?acc=GSE81152, accessed on 19 May 2025), containing whole blood-derived miRNA data. This selection allowed a cross-disorder and cross-tissue comparison between central (brain regions) and peripheral (serum and blood) miRNA signatures, aiming to identify both shared and disorder-specific molecular markers potentially relevant to the differential diagnosis of AD and MDD.

### 4.2. Data Download and Normalization

The identified datasets were downloaded from the NCBI Gene Expression Omnibus (GEO) database ([https://www.ncbi.nlm.nih.gov/geo/], accessed on 19 May 2025) in their raw or series matrix formats, depending on availability. All subsequent analyses were performed using the R programming language (version 4.5.1; R Core Team) and Bioconductor packages GEOquery (v2.76.0) and limma (v3.64.3) from Bioconductor release 3.21. Prior to differential expression analysis, each dataset underwent log_2_ transformation to stabilize variance and quantile normalization to ensure comparable expression distributions across samples. 

### 4.3. Differential Expression Analysis

Differential expression was assessed using the limma package with empirical Bayes moderation. The primary significance threshold was *p* < 0.05 and |log_2_ fold change| ≥ 1. However, for some datasets, no miRNAs met these strict criteria. In such cases, thresholds were relaxed to |log_2_ fold change| ≥ 0.2 and significance level < 1 (consistent with volcano plot parameters) to identify potential candidates for comparative analysis. This adjustment enabled the selection of a consistent set of candidate miRNAs across all datasets.

### 4.4. Common miRNA Identification

After independent differential expression analysis in each dataset, a panel of 10 overlapping miRNAs was selected based on shared presence between AD and MDD cohorts. This list included: hsa-miR-24-3p, hsa-miR-125a-3p, hsa-miR-664b-3p, hsa-miR-4443, hsa-miR-6131, hsa-miR-22-3p, hsa-miR-93-3p, hsa-miR-490-3p, hsa-miR-1299, and hsa-miR-1202.

### 4.5. Volcano Plot Visualization

Volcano plots for each selected dataset were generated using the GEO2R tool (NCBI Gene Expression Omnibus, https://www.ncbi.nlm.nih.gov/geo/; National Center for Biotechnology Information, Bethesda, MD, USA; accessed on 19 May 2025; version not specified by NCBI GEO2R). The plots simultaneously display the statistical significance and magnitude of change for differentially expressed miRNAs, with the horizontal axis representing log_2_ fold change (log_2_FC) and the vertical axis representing −log_10_(*p*-value). Significance thresholds were set at |log_2_FC| ≥ 0.2 and *p* < 1, which allowed the identification of candidate miRNAs in datasets where no significant results were obtained under the *p* < 0.05 criterion.

### 4.6. Target Gene Prediction and Functional Enrichment Analysis

Among the differentially expressed miRNAs identified in AD and MDD, *hsa-miR-1202* emerged as a shared miRNA of interest. Target gene prediction for *hsa-miR-1202* was conducted using miRDB (version 6.0; MirTarget v4; miRBase 22; Liu Lab, Washington University School of Medicine; http://mirdb.org, accessed on 6 August 2025), retaining only high-confidence targets with a prediction score ≥80, resulting in a total of 81 genes.

Gene Ontology (GO) enrichment analysis of these targets was performed using the DIANA-miRPath v4.0 platform (DIANA-Lab, University of Thessaly, Larissa, Greece; http://62.217.122.229:3838/app/miRPathv4/, accessed on 6 August 2025), enabling the identification of biological processes and molecular functions potentially regulated by *hsa-miR-1202*.

To further explore the involvement of these target genes in molecular pathways, KEGG 2021 Human pathway enrichment analysis was carried out using the Enrichr platform (Ma’ayan Laboratory, Icahn School of Medicine at Mount Sinai, New York, NY, USA; https://maayanlab.cloud/Enrichr, accessed on 6 August 2025). The enrichment results indicated significant associations with multiple biological pathways, including renal cell carcinoma, Ras signaling, ErbB signaling, axon guidance, MAPK signaling, T cell receptor signaling, focal adhesion, leukocyte transendothelial migration, regulation of actin cytoskeleton, and glycosaminoglycan biosynthesis. These enriched pathways were subsequently integrated into a miRNA–mRNA–pathway regulatory network for visualization and interpretation.

### 4.7. Integrated Network Analysis

High-confidence miRNA–mRNA interactions, derived from the intersection of predictions from miRDB (http://mirdb.org; accessed on 6 August 2025), TargetScan (TargetScanHuman Release 8.0; Bartel Lab, Whitehead Institute for Biomedical Research, Cambridge, MA, USA; https://www.targetscan.org/vert_80/; accessed on 6 August 2025), and miRTarBase v10.0 (School of Medicine, The Chinese University of Hong Kong, Shenzhen, China; https://mirtarbase.cuhk.edu.cn/; accessed on 6 August 2025), together with significantly enriched GO Biological Process and KEGG pathway terms obtained from DIANA-miRPath v4.0 (http://62.217.122.229:3838/app/miRPathv4/; accessed on 6 August 2025), were imported into Cytoscape (Cytoscape Consortium; v3.10.3; https://cytoscape.org; accessed on 6 August 2025) to construct comprehensive regulatory networks. Network ontology grouping was further examined using the ClueGO app v2.5.10.

These integrated networks represented the global interactions between differentially expressed miRNAs, their predicted target genes, and associated functional pathways across AD and MDD datasets. Node size and color were mapped to enrichment significance, while edge styles reflected interaction type (inhibitory vs. associative). Functional modules and hub nodes were identified through network topology analysis, and biological grouping was further examined using the ClueGO plugin (kappa score = 0.4), enabling the identification of disease-specific and shared regulatory clusters with potential diagnostic relevance.

### 4.8. Network Construction and Visualization (hsa-miR-1202 Case Study)

Predicted target genes of hsa-miR-1202 were retrieved from miRDB (http://mirdb.org; score cutoff ≥ 80; accessed on 6 August 2025), yielding 81 candidate genes. KEGG pathway enrichment was subsequently performed using the Enrichr platform (https://maayanlab.cloud/Enrichr; accessed on 6 August 2025), and only genes significantly associated with Gene Ontology (GO) Biological Process terms (*p* < 0.05) were retained, resulting in 51 genes. For each retained gene, the most significant GO term (minimum *p*-value) was selected to represent its primary functional annotation.

A bipartite network was then constructed, containing miRNA→gene (“targets”) edges and GO→gene (“annotates”) edges. The network was imported into Cytoscape (v3.10.3; https://cytoscape.org; accessed on 6 August 2025) via a GraphML file containing node attributes (Type, *p*_value, −log10p). Visualization parameters were set as follows: node size mapped to −log10(p), miRNA→gene edges styled as inhibitory (red, blunt end), and GO→gene edges styled as neutral (gray, arrow). The Group Attributes Layout and yFiles Organic layout algorithms were applied to optimize network readability (yFiles Layout Algorithms for Cytoscape app; yWorks GmbH, Tübingen, Germany). This case study specifically illustrates the regulatory landscape and functional associations of hsa-miR-1202 without integrating other miRNAs from the dataset.

## 5. Conclusions

In conclusion, this integrative bioinformatics study underscores the significant potential of circulating miRNA profiling as a minimally invasive approach for differentiating AD from MDD, two conditions that often share overlapping clinical symptoms. The identification of both unique and commonly dysregulated miRNAs highlights distinct yet convergent molecular pathways, including those related to synaptic plasticity, neuroinflammation, neurotransmitter signaling, and cellular stress responses. Functional enrichment and network analyses further emphasize the biological plausibility of these miRNAs as regulators of key neuropsychiatric processes. Importantly, the robustness of these findings across multiple tissues and biofluids suggests that such biomarkers could be applicable in diverse clinical contexts. While the results provide a valuable foundation, future multicenter studies with larger, ethnically diverse cohorts and prospective validation are needed to translate these insights into practical diagnostic applications. Additionally, combining miRNA signatures with other omics data may refine disease stratification and inform the development of personalized therapeutic strategies for complex neuropsychiatric disorders.

## Figures and Tables

**Table 1:** Differentially Expressed miRNAs in Alzheimer’s Disease Brain Tissue (GSE157239).

miRNA	adj.P.Val	*p*.Value	logFC
hsa-miR-1299	0.947	0.083736	1.497876
hsa-miR-1202	0.947	0.030694	1.302053
hsa-miR-4492	0.947	0.014047	0.936836
hsa-miR-3162-5p	0.947	0.03052	0.886615
hsa-miR-1247-3p	0.947	0.138094	0.861453
hsa-miR-7150	0.947	0.015806	0.848079
hsa-miR-1229-5p	0.947	0.072736	0.820246
hsa-miR-93-3p	0.947	0.274243	0.791748
hsa-miR-6132	0.947	0.154666	0.773815
hsa-miR-4443	0.947	0.035603	0.73685
hsa-miR-4286	0.106	1.59 × 10^−5^	−1.66659
hsa-miR-3651	0.947	0.017317	−1.3569
hsa-miR-664b-3p	0.947	0.021443	−1.15311
hsa-miR-4449	0.947	0.036094	−1.11271
hsa-miR-873-5p	0.947	0.113317	−0.9492
hsa-miR-490-3p	0.947	0.013405	−0.90274
hsa-miR-1246	0.947	0.109332	−0.85051
hsa-miR-339-5p	0.947	0.101191	−0.84158
hsa-miR-365b-5p	0.947	0.006537	−0.8342
hsa-miR-30e-3p	0.947	0.030222	−0.82802

**Table 2:** Top 10 Upregulated and Downregulated miRNAs in Serum Samples of AD Patients Compared to Controls (GSE120584 Dataset).

miRNA	adj.P.Val	*p*.Value	logFC
hsa-miR-208a-5p	3.5 × 10^−16^	3.01 × 10^−18^	0.592526
hsa-miR-6761-3p	2.98 × 10^−22^	1.16 × 10^−25^	0.461466
hsa-miR-3646	6.49 × 10^−11^	2.86 × 10^−12^	0.433593
hsa-miR-595	1.83 × 10^−13^	3.78 × 10^−15^	0.431681
hsa-miR-6754-3p	4.34 × 10^−17^	2.71 × 10^−19^	0.425993
hsa-miR-6736-3p	6.64 × 10^−17^	4.41 × 10^−19^	0.420703
hsa-miR-6762-3p	2.44 × 10^−16^	1.9 × 10^−18^	0.417395
hsa-miR-4435	1.82 × 10^−6^	1.9 × 10^−7^	0.415216
hsa-miR-6747-3p	3.49 × 10^−18^	1.08 × 10^−20^	0.414607
hsa-miR-6716-3p	4.76 × 10^−9^	2.9 × 10^−10^	0.405795
hsa-miR-125a-3p	1.32 × 10^−7^	1.07 × 10^−8^	−0.49493
hsa-miR-6131	0.000266	4.23 × 10^−5^	−0.4454
hsa-miR-24-3p	4.35 × 10^−5^	5.79 × 10^−6^	−0.4384
hsa-miR-125b-1-3p	0.000517	8.84 × 10^−5^	−0.42619
hsa-miR-22-3p	2.95 × 10^−5^	3.77 × 10^−6^	−0.41228
hsa-miR-516a-5p	3.79 × 10^−5^	4.97 × 10^−6^	−0.36696
hsa-miR-3184-5p	3.89 × 10^−20^	3.04 × 10^−23^	−0.35231
hsa-miR-520f-5p	4.14 × 10^−5^	5.48 × 10^−6^	−0.3521
hsa-miR-887-5p	0.000034	4.42 × 10^−6^	−0.34
hsa-miR-6070	8.36 × 10^−5^	1.19 × 10^−5^	−0.33521

**Table 3:** Top 10 Upregulated and Downregulated miRNAs in MDD (GSE81152, whole blood).

miRNA	adj.P.Val	*p*.Value	logFC
hsa-miR-4445-3p	0.996	0.16099	0.7765884
hsa-miR-5579-5p	0.996	0.02361	0.7670802
hsa-miR-3129-3p	0.996	0.04917	0.7365824
hsa-miR-5695	0.996	0.10672	0.7277248
hsa-miR-3160-3p	0.996	0.14556	0.7177084
hsa-miR-4666a-3p	0.996	0.203	0.6594012
hsa-miR-4494	0.996	0.05509	0.654006
hsa-miR-4528	0.996	0.08772	0.652799
hsa-miR-137	0.996	0.09897	0.6433831
hsa-miR-3978	0.996	0.20371	0.6253926
hsa-let-7b-5p	0.996	0.01956	−0.3386143
hsa-miR-539-3p	0.996	0.23933	−0.3261406
hsa-miR-1185-2-3p	0.996	0.00275	−0.3214392
hsa-let-7c	0.996	0.02448	−0.3046885
hsa-miR-4310	0.996	0.01135	−0.2960535
hsa-miR-4730	0.996	0.35984	−0.2575695
hsa-miR-4429	0.996	0.0182	−0.248767
hsa-miR-1277-3p	0.996	0.09126	−0.2455134
hsa-miR-320b	0.996	0.03201	−0.2401544
hsa-miR-320d	0.996	0.02507	−0.2397299

**Table 4:** Top 10 upregulated and top 10 downregulated miRNAs identified from the prefrontal cortex of individuals with MDD, based on the GSE58105 dataset.

miRNA	adj.P.Val	*p*.Value	logFC
hsa-miR-451	0.975	0.32773	0.457753
hsa-miR-1281	0.96	0.01357	0.386582
hsa-miR-1825	0.96	0.01916	0.368593
hsa-miR-137	0.975	0.34946	0.307467
hsa-miR-940	0.968	0.21643	0.299043
hsa-miR-103	0.975	0.32593	0.286291
hsa-let-7i	0.975	0.38183	0.2752
hsa-miR-149	0.96	0.05461	0.259892
hsa-miR-425*	0.96	0.03003	0.254973
hsa-miR-766	0.96	0.05497	0.254391
hsa-miR-1202	0.96	0.06943	−0.8499
hsa-miR-575	0.968	0.18356	−0.53419
hsa-miR-1225-5p	0.968	0.20087	−0.50029
hsa-miR-1275	0.968	0.16304	−0.4578
hsa-miR-1274a	0.975	0.39305	−0.45505
hsa-miR-1826	0.96	0.05979	−0.4539
hsa-miR-1260	0.975	0.38413	−0.41749
hsa-miR-1207-5p	0.975	0.37012	−0.40515
hsa-miR-513a-5p	0.96	0.08625	−0.39608
hsa-miR-188-5p	0.968	0.18532	−0.39594

**Table 5:** Differentially Expressed miRNAs in AD and MDD across Multiple Tissues ( ↓: downregulation; ↑: upregulation; NA: not applicable).

miRNA	GSE157239 (TC, AD)	GSE120584 (Serum, AD)	GSE58105 (vlPFC, MDD)	GSE81152 (Blood, MDD)	Expression Concordance
hsa-miR-24-3p	Down	Down	Down	Down	Conserved (↓)
hsa-miR-125a-3p	NA	Down	NA	Down	Partial (↓ in 2)
hsa-miR-664b-3p	Down	NA	Down	NA	Partial (↓ in 2)
hsa-miR-4443	Up	NA	Up	NA	Partial (↑ in 2)
hsa-miR-6131	NA	Down	Down	NA	Partial (↓ in 2)
hsa-miR-22-3p	NA	Down	NA	Down	Partial (↓ in 2)
hsa-miR-93-3p	Up	NA	NA	NA	NA
hsa-miR-490-3p	Down	NA	NA	NA	NA
hsa-miR-1299	Up	NA	NA	NA	NA
hsa-miR-1202	Up	NA	NA	NA	NA

**Table 6:** Top 10 GO and KEGG Pathways for AD and MDD.

Group	Type	Pathway	miRNAs (*n*)	*p*-Value	FDR
AD	GO	protein binding	21	7.18 × 10^−172^	1.346 × 10^−167^
AD	GO	nucleus	21	2.72 × 10^−152^	2.549 × 10^−148^
AD	GO	nucleoplasm	21	2.877 × 10^−151^	1.797 × 10^−147^
AD	GO	cytoplasm	21	3.724 × 10^−134^	1.745 × 10^−130^
AD	GO	cytosol	21	8.54 × 10^−134^	3.201 × 10^−130^
AD	GO	RNA binding	21	5.3277 × 10^−80^	1.6641 × 10^−76^
AD	GO	cadherin binding	19	1.1094 × 10^−47^	2.9701 × 10^−44^
AD	GO	protein-containing complex	19	1.5845 × 10^−38^	3.7119 × 10^−35^
AD	GO	viral process	19	1.7127 × 10^−37^	3.5665 × 10^−34^
AD	GO	nucleotide binding	21	3.7218 × 10^−36^	6.975 × 10^−33^
AD	KEGG	Cell cycle	16	6.359 × 10^−16^	2.1684 × 10^−13^
AD	KEGG	Pathways in cancer	19	7.0558 × 10^−14^	1.203 × 10^−11^
AD	KEGG	Shigellosis	19	1.2991 × 10^−12^	1.4766 × 10^−10^
AD	KEGG	PI3K-Akt signaling pathway	20	2.0311 × 10^−12^	1.7315 × 10^−10^
AD	KEGG	Proteoglycans in cancer	17	5.727 × 10^−11^	3.9058 × 10^−9^
AD	KEGG	Focal adhesion	16	2.7194 × 10^−10^	1.5455 × 10^−8^
AD	KEGG	Chronic myeloid leukemia	15	4.5103 × 10^−10^	2.1972 × 10^−8^
AD	KEGG	Salmonella infection	20	1.156 × 10^−9^	4.9274 × 10^−8^
AD	KEGG	MAPK signaling pathway	18	2.8926 × 10^−9^	1.096 × 10^−7^
AD	KEGG	Adherens junction	16	7.3426 × 10^−9^	2.5038 × 10^−7^
MDD	GO	protein binding	17	3.343 × 10^−133^	6.264 × 10^−129^
MDD	GO	nucleus	17	4.77 × 10^−119^	4.47 × 10^−115^
MDD	GO	nucleoplasm	17	3.695 × 10^−114^	2.308 × 10^−110^
MDD	GO	cytosol	17	1.0229 × 10^−86^	4.7928 × 10^−83^
MDD	GO	cytoplasm	16	9.4339 × 10^−78^	3.536 × 10^−74^
MDD	GO	RNA binding	16	4.3253 × 10^−56^	1.351 × 10^−52^
MDD	GO	chromatin binding	17	8.1889 × 10^−34^	2.1924 × 10^−30^
MDD	GO	viral process	16	4.5159 × 10^−32^	1.0579 × 10^−28^
MDD	GO	ATP binding	17	5.5876 × 10^−29^	1.1635 × 10^−25^
MDD	GO	nucleotide binding	16	1.8043 × 10^−28^	3.3815 × 10^−25^
MDD	KEGG	Hippo signaling pathway	16	4.5 × 10^−13^	1.5345 × 10^−10^
MDD	KEGG	Protein processing in endoplasmic reticulum	15	8.1274 × 10^−11^	1.3857 × 10^−8^
MDD	KEGG	Proteoglycans in cancer	15	1.5864 × 10^−9^	1.8032 × 10^−7^
MDD	KEGG	Cell cycle	13	4.0933 × 10^−9^	2.7916 × 10^−7^
MDD	KEGG	Adherens junction	16	3.5925 × 10^−9^	2.7916 × 10^−7^
MDD	KEGG	Pathways in cancer	14	6.9102 × 10^−9^	3.9273 × 10^−7^
MDD	KEGG	Ubiquitin mediated proteolysis	16	8.84 × 10^−9^	4.3064 × 10^−7^
MDD	KEGG	Alzheimer disease	16	3.8316 × 10^−8^	1.6332 × 10^−6^
MDD	KEGG	Amyotrophic lateral sclerosis	15	6.1199 × 10^−8^	2.3188 × 10^−6^
MDD	KEGG	Hepatitis B	16	1.0497 × 10^−7^	3.5795 × 10^−6^

**Table 7:** Top 51 predicted target genes of hsa-miR-1202 and their most significantly associated GO Biological Process terms.

Gene	GO_ID	GO_Term	*p*_Value	−log10(p)
*ACTG1*	GO:0010468	Regulation of Gene Expression (GO:0010468)	0.000566615	3.246711667
*ZNF195*	GO:0010468	Regulation of Gene Expression (GO:0010468)	0.000566615	3.246711667
*MED23*	GO:0010468	Regulation of Gene Expression (GO:0010468)	0.000566615	3.246711667
*NDFIP2*	GO:0010468	Regulation of Gene Expression (GO:0010468)	0.000566615	3.246711667
*DMTF1*	GO:0010468	Regulation of Gene Expression (GO:0010468)	0.000566615	3.246711667
*DICER1*	GO:0010468	Regulation of Gene Expression (GO:0010468)	0.000566615	3.246711667
*RRP1B*	GO:0010468	Regulation of Gene Expression (GO:0010468)	0.000566615	3.246711667
*KDM6A*	GO:0010468	Regulation of Gene Expression (GO:0010468)	0.000566615	3.246711667
*SRRM4*	GO:0010468	Regulation of Gene Expression (GO:0010468)	0.000566615	3.246711667
*CDH5*	GO:0010468	Regulation of Gene Expression (GO:0010468)	0.000566615	3.246711667
*ZBTB34*	GO:0010468	Regulation of Gene Expression (GO:0010468)	0.000566615	3.246711667
*BCL6B*	GO:0010468	Regulation of Gene Expression (GO:0010468)	0.000566615	3.246711667
*SRPK2*	GO:0010468	Regulation of Gene Expression (GO:0010468)	0.000566615	3.246711667
*SLC38A1*	GO:0001504	Neurotransmitter Uptake (GO:0001504)	0.000870149	3.060406388
*SLC18A2*	GO:0001504	Neurotransmitter Uptake (GO:0001504)	0.000870149	3.060406388
*ZDHHC18*	GO:0018230	peptidyl-L-cysteine S-palmitoylation (GO:0018230)	0.001428394	2.845152025
*ZDHHC21*	GO:0018230	peptidyl-L-cysteine S-palmitoylation (GO:0018230)	0.001428394	2.845152025
*DNAJA1*	GO:1904950	Negative Regulation of Establishment of Protein Localization (GO:1904950)	0.001643823	2.784144975
*LUZP1*	GO:0003382	Epithelial Cell Morphogenesis (GO:0003382)	0.00211799	2.674076155
*ZNF365*	GO:0045687	Positive Regulation of Glial Cell Differentiation (GO:0045687)	0.00211799	2.674076155
*CTNND1*	GO:0002042	Cell Migration Involved in Sprouting Angiogenesis (GO:0002042)	0.00355059	2.449699495
*PLPPR4*	GO:0048812	Neuron Projection Morphogenesis (GO:0048812)	0.003805383	2.419601683
*EFNA5*	GO:0007409	Axonogenesis (GO:0007409)	0.008869378	2.052106841
*SOS1*	GO:0007409	Axonogenesis (GO:0007409)	0.008869378	2.052106841
*COLEC10*	GO:0002699	Positive Regulation of Immune Effector Process (GO:0002699)	0.009335188	2.029876909
*HLA-DRA*	GO:0002699	Positive Regulation of Immune Effector Process (GO:0002699)	0.009335188	2.029876909
*ARID4B*	GO:1902459	Positive Regulation of Stem Cell Population Maintenance (GO:1902459)	0.011437859	1.941655261
*BICRAL*	GO:1902459	Positive Regulation of Stem Cell Population Maintenance (GO:1902459)	0.011437859	1.941655261
*MCTP2*	GO:0050804	Modulation of Chemical Synaptic Transmission (GO:0050804)	0.011957518	1.922358969
*CLIC4*	GO:0030334	Regulation of Cell Migration (GO:0030334)	0.013100821	1.882701502
*PPP2R3A*	GO:0030334	Regulation of Cell Migration (GO:0030334)	0.013100821	1.882701502
*LRRC8C*	GO:0006820	Monoatomic Anion Transport (GO:0006820)	0.015565293	1.807842695
*TOX*	GO:0021895	Cerebral Cortex Neuron Differentiation (GO:0021895)	0.020088479	1.697052943
*CNST*	GO:0042998	Positive Regulation of Golgi to Plasma Membrane Protein Transport (GO:0042998)	0.020088479	1.697052943
*SELENOT*	GO:0060124	Positive Regulation of Growth Hormone Secretion (GO:0060124)	0.020088479	1.697052943
*FMO5*	GO:0070995	NADPH Oxidation (GO:0070995)	0.020088479	1.697052943
*NOD1*	GO:0002579	Positive Regulation of Antigen Processing and Presentation (GO:0002579)	0.020088479	1.697052943
*EGLN2*	GO:0019471	4-Hydroxyproline Metabolic Process (GO:0019471)	0.020088479	1.697052943
*EIF4E2*	GO:0045824	Negative Regulation of Innate Immune Response (GO:0045824)	0.022391421	1.649918343
*CCR5*	GO:0014808	Release of Sequestered Calcium Ion Into Cytosol by Sarcoplasmic Reticulum (GO:0014808)	0.024058097	1.618738721
*RRN3*	GO:0006352	DNA-templated Transcription Initiation (GO:0006352)	0.026188578	1.581888083
*LILRA5*	GO:0050731	Positive Regulation of Peptidyl-Tyrosine Phosphorylation (GO:0050731)	0.02697727	1.569001997
*NDST3*	GO:0015014	Heparan Sulfate Proteoglycan Biosynthetic Process, Chain Elongation (GO:0015014)	0.028011834	1.552658455
*NR2C1*	GO:0000122	Negative Regulation of Transcription by RNA Polymerase II (GO:0000122)	0.028753364	1.541311336
*MAX*	GO:0000122	Negative Regulation of Transcription by RNA Polymerase II (GO:0000122)	0.028753364	1.541311336
*ZBTB43*	GO:0006357	Regulation of Transcription by RNA Polymerase II (GO:0006357)	0.03528067	1.452463176
*ZFHX4*	GO:0006357	Regulation of Transcription by RNA Polymerase II (GO:0006357)	0.03528067	1.452463176
*DENND6A*	GO:2000049	Positive Regulation of Cell-Cell Adhesion Mediated by Cadherin (GO:2000049)	0.035871912	1.445245477
*SYNJ2BP*	GO:0030336	Negative Regulation of Cell Migration (GO:0030336)	0.038770418	1.41149952
*NUMBL*	GO:0021846	Cell Proliferation in Forebrain (GO:0021846)	0.039778377	1.400352938
*DNAJB6*	GO:0090084	Negative Regulation of Inclusion Body Assembly (GO:0090084)	0.039778377	1.400352938

## Data Availability

Data are contained within the article and Supplementary Materials.

## Supplementary Materials

The following supporting information can be downloaded at: https://www.mdpi.com/article/10.3390/ijms26178218/s1.
