# Supplementary material for: The Role of miRNAs in the Differential Diagnosis of Alzheimer’s Disease and Major Depression: A Bioinformatics-Based Approach
Source: Int J Mol Sci. 2025 Aug 24;26(17):8218. doi: 10.3390/ijms26178218 (PMC12428279; doi:10.3390/ijms26178218)
Supplement: Supplementary file 1 [file ijms-26-08218-s001.zip › ijms-3765769-supplementary.pdf]

Supplementary Table S1. Enrichr-based KEGG enrichment for hsa-miR-1202 predicted targets (miRDB ≥80)

Term	Overlap	P-value	Adjusted P-value	Old P-value	Old Adjusted P-value	Odds Ratio	Combined Score	Genes
Renal cell carcinoma	4/69	0,000177	0,024383023	0	0	15,86733267	137,1115553	EGLN2;SOS1;PAK2;PAK5
Ras signaling pathway	5/232	0,00252	0,173882547	0	0	5,707164387	34,14871383	RALBP1;SOS1;EFNA5;PAK2;PAK5
ErbB signaling pathway	3/85	0,004979	0,177864771	0	0	9,304409006	49,33694019	SOS1;PAK2;PAK5
Axon guidance	4/182	0,006404	0,177864771	0	0	5,761272435	29,0994664	ABLIM1;EFNA5;PAK2;PAK5
MAPK signaling pathway	5/294	0,006848	0,177864771	0	0	4,468676015	22,2710378	MAX;SOS1;EFNA5;PAK2;MAP4K3
T cell receptor signaling pathway	3/104	0,00869	0,177864771	0	0	7,546839299	35,81416313	SOS1;PAK2;PAK5
Focal adhesion	4/201	0,009022	0,177864771	0	0	5,2006065	24,48484683	SOS1;PAK2;PAK5;ACTG1
Leukocyte transendothelial migration	3/114	0,011149	0,182260499	0	0	6,863478863	30,86080759	CDH5;CTNND1;ACTG1
Regulation of actin cytoskeleton	4/218	0,011887	0,182260499	0	0	4,783347494	21,20145788	SOS1;PAK2;PAK5;ACTG1
Glycosaminoglycan biosynthesis	2/53	0,019535	0,269578995	0	0	9,862496897	38,81447203	NDST3;CHSY3
Notch signaling pathway	2/59	0,023881	0,283362125	0	0	8,821674439	32,94618603	TLE3;NUMBL
Viral myocarditis	2/60	0,02464	0,283362125	0	0	8,669140113	32,105091	HLA-DRA;ACTG1
Adherens junction	2/71	0,033623	0,340017907	0	0	7,283067327	24,70811144	CTNND1;ACTG1
Non-small cell lung cancer	2/72	0,034495	0,340017907	0	0	7,178661844	24,17021954	EML4;SOS1
B cell receptor signaling pathway	2/81	0,042723	0,371663611	0	0	6,357955456	20,04680786	SOS1;LILRA5
Transcriptional misregulation in cancer	3/192	0,043091	0,371663611	0	0	4,015059015	12,62507631	MAX;CDK14;KDM6A
Rap1 signaling pathway	3/210	0,053655	0,403733104	0	0	3,662578967	10,71372088	CTNND1;EFNA5;ACTG1
Human immunodeficiency virus 1 infection	3/212	0,054899	0,403733104	0	0	3,627162311	10,5269749	CCR5;PAK2;PAK5
PI3K-Akt signaling pathway	4/354	0,055586	0,403733104	0	0	2,904489796	8,393440968	PPP2R3A;SOS1;EIF4E2;EFNA5
Pathways in cancer	5/531	0,064281	0,443541777	0	0	2,425580348	6,65696814	EML4;RALBP1;EGLN2;MAX;SOS1
Other glycan degradation	1/18	0,070472	0,453129567	0	0	14,63382353	38,81670264	MAN2B2
HIF-1 signaling pathway	2/109	0,072238	0,453129567	0	0	4,687566544	12,31793302	EGLN2;EIF4E2
Toxoplasmosis	2/112	0,075707	0,454240065	0	0	4,559033372	11,76635868	HLA-DRA;CCR5
AMPK signaling pathway	2/120	0,085207	0,489942459	0	0	4,248229994	10,46197658	HNF4A;PPP2R3A
Dopaminergic synapse	2/132	0,100086	0,513777052	0	0	3,853748783	8,870284845	PPP2R3A;SLC18A2
Maturity onset diabetes of the young	1/26	0,100197	0,513777052	0	0	9,947	22,88422332	HNF4A
Insulin signaling pathway	2/137	0,106486	0,513777052	0	0	3,7100797	8,309621578	SOS1;EIF4E2

Supplementary Table S1. Enrichr-based KEGG enrichment for hsa-miR-1202 predicted targets (miRDB ≥80)

Fluid shear stress and atherosclerosis	2/139	0,109077	0,513777052	0	0	3,655548369	8,099613945	CDH5;ACTG1
Asthma	1/31	0,118296	0,513777052	0	0	8,287083333	17,6893023	HLA-DRA
Cell adhesion molecules	2/148	0,120937	0,513777052	0	0	3,428645743	7,242971614	CDH5;HLA-DRA
Phagosome	2/152	0,126307	0,513777052	0	0	3,336540084	6,903431681	HLA-DRA;ACTG1
mTOR signaling pathway	2/154	0,129014	0,513777052	0	0	3,29230513	6,742104888	SOS1;EIF4E2
MicroRNAs in cancer	3/310	0,131101	0,513777052	0	0	2,457028314	4,992159315	DICER1;SOS1;EFNA5
Allograft rejection	1/38	0,143032	0,513777052	0	0	6,716891892	13,06223267	HLA-DRA
Hepatocellular carcinoma	2/168	0,148322	0,513777052	0	0	3,012505719	5,74898065	SOS1;ACTG1
Influenza A	2/172	0,153944	0,513777052	0	0	2,94102755	5,503160748	HLA-DRA;ACTG1
Graft-versus-host disease	1/42	0,156858	0,513777052	0	0	6,060365854	11,22629749	HLA-DRA
Type I diabetes mellitus	1/43	0,16028	0,513777052	0	0	5,91577381	10,83078675	HLA-DRA
Fat digestion and absorption	1/43	0,16028	0,513777052	0	0	5,91577381	10,83078675	ABCA1
ABC transporters	1/45	0,167083	0,513777052	0	0	5,646306818	10,10274	ABCA1
Proteasome	1/46	0,170464	0,513777052	0	0	5,520555556	9,767144596	PSMD12
Alcoholism	2/186	0,173929	0,513777052	0	0	2,715327463	4,749401114	SOS1;SLC18A2
Intestinal immune network for IgA production	1/48	0,177185	0,513777052	0	0	5,285106383	9,146197104	HLA-DRA
Cocaine addiction	1/49	0,180525	0,513777052	0	0	5,174739583	8,858551176	SLC18A2
Chemokine signaling pathway	2/192	0,182622	0,513777052	0	0	2,628780813	4,469818992	CCR5;SOS1
Vibrio cholerae infection	1/50	0,183852	0,513777052	0	0	5,068877551	8,584760541	ACTG1
Cholesterol metabolism	1/50	0,183852	0,513777052	0	0	5,068877551	8,584760541	ABCA1
Pathogenic Escherichia coli infection	2/197	0,189915	0,513777052	0	0	2,560727037	4,253828376	PAK2;ACTG1
Autoimmune thyroid disease	1/53	0,193754	0,513777052	0	0	4,775721154	7,837758584	HLA-DRA
Epstein-Barr virus infection	2/202	0,197248	0,513777052	0	0	2,496075949	4,051865187	PSMD12;HLA-DRA
Proteoglycans in cancer	2/205	0,201665	0,513777052	0	0	2,458813993	3,936922754	SOS1;ACTG1
Endometrial cancer	1/58	0,209993	0,513777052	0	0	4,355701754	6,79786665	SOS1
Human cytomegalovirus infection	2/225	0,231372	0,513777052	0	0	2,236022024	3,27292973	CCR5;SOS1
Inflammatory bowel disease	1/65	0,232186	0,513777052	0	0	3,877929688	5,662617012	HLA-DRA
Acute myeloid leukemia	1/67	0,238413	0,513777052	0	0	3,760037879	5,390956777	SOS1
Fc epsilon RI signaling pathway	1/68	0,241508	0,513777052	0	0	3,703731343	5,262459243	SOS1

Supplementary Table S1. Enrichr-based KEGG enrichment for hsa-miR-1202 predicted targets (miRDB ≥80)

Thermogenesis	2/232	0,241846	0,513777052	0	0	2,167198679	3,07624115	SOS1;ACTG1
Amphetamine addiction	1/69	0,24459	0,513777052	0	0	3,649080882	5,138530367	SLC18A2
Epithelial cell signaling in Helicobacter pylori infection	1/70	0,24766	0,513777052	0	0	3,596014493	5,018949012	NOD1
Prolactin signaling pathway	1/70	0,24766	0,513777052	0	0	3,596014493	5,018949012	SOS1
Glioma	1/75	0,262826	0,513777052	0	0	3,352195946	4,479411738	SOS1
Shigellosis	2/246	0,262854	0,513777052	0	0	2,04139863	2,727631108	NOD1;ACTG1
Chronic myeloid leukemia	1/76	0,265823	0,513777052	0	0	3,307333333	4,381966173	SOS1
Gastric acid secretion	1/76	0,265823	0,513777052	0	0	3,307333333	4,381966173	ACTG1
Pancreatic cancer	1/76	0,265823	0,513777052	0	0	3,307333333	4,381966173	RALBP1
Pertussis	1/76	0,265823	0,513777052	0	0	3,307333333	4,381966173	NOD1
Salmonella infection	2/249	0,26736	0,513777052	0	0	2,016296828	2,659814607	NOD1;ACTG1
Parkinson disease	2/249	0,26736	0,513777052	0	0	2,016296828	2,659814607	PSMD12;SLC18A2
Arrhythmogenic right ventricular cardiomyopathy	1/77	0,268808	0,513777052	0	0	3,263651316	4,287649604	ACTG1
Bacterial invasion of epithelial cells	1/77	0,268808	0,513777052	0	0	3,263651316	4,287649604	ACTG1
Leishmaniasis	1/77	0,268808	0,513777052	0	0	3,263651316	4,287649604	HLA-DRA
Synaptic vesicle cycle	1/78	0,271781	0,513777052	0	0	3,221103896	4,196325643	SLC18A2
Antigen processing and presentation	1/78	0,271781	0,513777052	0	0	3,221103896	4,196325643	HLA-DRA
Colorectal cancer	1/86	0,295137	0,518265253	0	0	2,916764706	3,559373891	SOS1
Gap junction	1/88	0,300859	0,518265253	0	0	2,849425287	3,42247877	SOS1
GABAergic synapse	1/89	0,303704	0,518265253	0	0	2,816903409	3,356913201	SLC38A1
PD-L1 expression and PD-1 checkpoint pathway in cancer	1/89	0,303704	0,518265253	0	0	2,816903409	3,356913201	EML4
Hypertrophic cardiomyopathy	1/90	0,306536	0,518265253	0	0	2,78511236	3,293171728	ACTG1
Small cell lung cancer	1/92	0,312167	0,518265253	0	0	2,723626374	3,170890169	MAX
Th1 and Th2 cell differentiation	1/92	0,312167	0,518265253	0	0	2,723626374	3,170890169	HLA-DRA
GnRH signaling pathway	1/93	0,314966	0,518265253	0	0	2,69388587	3,112222607	SOS1
Rheumatoid arthritis	1/93	0,314966	0,518265253	0	0	2,69388587	3,112222607	HLA-DRA
Staphylococcus aureus infection	1/95	0,320529	0,518265253	0	0	2,636303191	2,99953725	HLA-DRA
Dilated cardiomyopathy	1/96	0,323294	0,518265253	0	0	2,608421053	2,945408908	ACTG1

Supplementary Table S1. Enrichr-based KEGG enrichment for hsa-miR-1202 predicted targets (miRDB ≥80)

Prostate cancer	1/97	0,326048	0,518265253	0	0	2,581119792	2,892687258	SOS1
Choline metabolism in cancer	1/98	0,328791	0,518265253	0	0	2,554381443	2,841323066	SOS1
mRNA surveillance pathway	1/98	0,328791	0,518265253	0	0	2,554381443	2,841323066	PPP2R3A
Hematopoietic cell lineage	1/99	0,331523	0,518265253	0	0	2,528188776	2,791269281	HLA-DRA
Viral protein interaction with cytokine and cytokine receptor	1/100	0,334244	0,518265253	0	0	2,502525253	2,742480916	CCR5
Longevity regulating pathway	1/102	0,339652	0,520800278	0	0	2,452722772	2,648530176	EIF4E2
Th17 cell differentiation	1/107	0,352985	0,53342965	0	0	2,336438679	2,433001258	HLA-DRA
Drug metabolism	1/108	0,35562	0,53342965	0	0	2,314485981	2,392931293	FMO5
Serotonergic synapse	1/113	0,368634	0,542129867	0	0	2,210602679	2,206072088	SLC18A2
Glutamatergic synapse	1/114	0,371206	0,542129867	0	0	2,190929204	2,171208285	SLC38A1
Sphingolipid signaling pathway	1/119	0,383909	0,542129867	0	0	2,097563559	2,008101058	PPP2R3A
Growth hormone synthesis, secretion and action	1/119	0,383909	0,542129867	0	0	2,097563559	2,008101058	SOS1
Neurotrophin signaling pathway	1/119	0,383909	0,542129867	0	0	2,097563559	2,008101058	SOS1
Human papillomavirus infection	2/331	0,3887	0,542129867	0	0	1,507444885	1,424454884	PPP2R3A;SOS1
Thyroid hormone signaling pathway	1/121	0,388919	0,542129867	0	0	2,062395833	1,947692665	ACTG1
Platelet activation	1/124	0,396359	0,546975475	0	0	2,011788618	1,861779222	ACTG1
Osteoclast differentiation	1/127	0,403709	0,548513412	0	0	1,96359127	1,781095258	LILRA5
Relaxin signaling pathway	1/129	0,40856	0,548513412	0	0	1,932714844	1,730003139	SOS1
FoxO signaling pathway	1/131	0,413372	0,548513412	0	0	1,902788462	1,680935379	SOS1
Natural killer cell mediated cytotoxicity	1/131	0,413372	0,548513412	0	0	1,902788462	1,680935379	SOS1
Systemic lupus erythematosus	1/135	0,422881	0,551455422	0	0	1,845615672	1,588456661	HLA-DRA
Yersinia infection	1/137	0,427578	0,551455422	0	0	1,818290441	1,544854332	ACTG1
Estrogen signaling pathway	1/137	0,427578	0,551455422	0	0	1,818290441	1,544854332	SOS1
Amyotrophic lateral sclerosis	2/364	0,435286	0,553453632	0	0	1,367718022	1,137602375	PSMD12;ACTG1
Apoptosis	1/142	0,439156	0,553453632	0	0	1,753368794	1,442849848	ACTG1
Spinocerebellar ataxia	1/143	0,441443	0,553453632	0	0	1,740933099	1,423571133	PSMD12
Breast cancer	1/147	0,450502	0,553453632	0	0	1,692893836	1,349901015	SOS1
Phospholipase D signaling pathway	1/148	0,452744	0,553453632	0	0	1,681292517	1,332303946	SOS1

Supplementary Table S1. Enrichr-based KEGG enrichment for hsa-miR-1202 predicted targets (miRDB ≥80)

Gastric cancer	1/149	0,454977	0,553453632	0	0	1,669847973	1,315019619	SOS1
Adrenergic signaling in cardiomyocytes	1/150	0,457201	0,553453632	0	0	1,658557047	1,298040713	PPP2R3A
Oxytocin signaling pathway	1/154	0,466007	0,559208618	0	0	1,614869281	1,233040276	ACTG1
Hepatitis C	1/157	0,472519	0,562134914	0	0	1,583573718	1,187168622	SOS1
Hepatitis B	1/162	0,483199	0,562795128	0	0	1,534006211	1,115724599	SOS1
JAK-STAT signaling pathway	1/162	0,483199	0,562795128	0	0	1,534006211	1,115724599	SOS1
Hippo signaling pathway	1/163	0,485309	0,562795128	0	0	1,524459877	1,102138455	ACTG1
Wnt signaling pathway	1/166	0,491588	0,565326564	0	0	1,496515152	1,062695864	TLE3
Tight junction	1/169	0,497792	0,567707541	0	0	1,469568452	1,025130885	ACTG1
Protein processing in endoplasmic reticulum	1/171	0,501886	0,567707541	0	0	1,452132353	1,001073216	DNAJA1
Tuberculosis	1/180	0,519906	0,58078899	0	0	1,37849162	0,901680478	HLA-DRA
NOD-like receptor signaling pathway	1/181	0,521868	0,58078899	0	0	1,370763889	0,891462438	NOD1
RNA transport	1/186	0,53156	0,586842642	0	0	1,333378378	0,842613151	EIF4E2
Neutrophil extracellular trap formation	1/189	0,537282	0,588451883	0	0	1,311901596	0,814995116	ACTG1
Kaposi sarcoma-associated herpesvirus infection	1/193	0,544804	0,591991702	0	0	1,284309896	0,779998979	CCR5
Viral carcinogenesis	1/203	0,563084	0,607075158	0	0	1,220111386	0,700741811	CCR5
Lipid and atherosclerosis	1/215	0,584065	0,624814226	0	0	1,150992991	0,618937495	ABCA1
Human T-cell leukemia virus 1 infection	1/219	0,590836	0,627194723	0	0	1,129644495	0,594438655	HLA-DRA
Herpes simplex virus 1 infection	2/498	0,602536	0,63473237	0	0	0,99137403	0,502238208	ZNF195;HLA-DRA
Chemical carcinogenesis	1/239	0,623088	0,651409743	0	0	1,033665966	0,488994489	SOS1
Endocytosis	1/252	0,642691	0,666852484	0	0	0,979482072	0,433020188	CCR5
Prion disease	1/273	0,672252	0,692319375	0	0	0,902895221	0,358559366	PSMD12
Cytokine-cytokine receptor interaction	1/295	0,700631	0,716200358	0	0	0,834396259	0,296856684	CCR5
Huntington disease	1/306	0,713896	0,724394234	0	0	0,803852459	0,270912999	PSMD12
Alzheimer disease	1/369	0,779412	0,785100735	0	0	0,664096467	0,165503468	PSMD12
Pathways of neurodegeneration	1/475	0,857855	0,857854726	0	0	0,512790084	0,078621237	PSMD12
